# Sociodemographic predictors of non-communicable diseases risk-related knowledge and behaviours: a cross-sectional study of in-school adolescents in a southern Nigerian State

**DOI:** 10.11604/pamj.2023.45.184.37654

**Published:** 2023-08-25

**Authors:** Patrick Oyibo, Ejiroghene Martha Umuerri, Mamodesan Tudjegbe Okumagba, Iyabo Aduke Oyibo

**Affiliations:** 1 Health Services Research and Management Division, School of Health and Psychological Sciences City, University of London, London, United Kingdom,; 2 Department of Community Medicine, Faculty of Clinical Medicine, College of Health Sciences, Delta State University, Abraka, Nigeria,; 3 Department of Medicine, Faculty of Clinical Medicine, College of Health Sciences, Delta State University, Abraka, Nigeria,; 4 Department of Pediatrics, Princess of Wales Hospital, Bridgend, United Kingdom

**Keywords:** Non-communicable diseases, non-communicable chronic diseases, non-infectious diseases, risk behaviours, lifestyle

## Abstract

**Introduction:**

the adolescence period is a significant phase in development of non-communicable diseases. Public health interventions that reduce risky behaviors among adolescents are beneficial across the life course. This study assessed the level of non-communicable diseases (NCDs´) risk-related knowledge, the prevalence of NCDs´ risk behavior, and the sociodemographic predictors of NCDs´ risk-related knowledge and behaviors among in-school adolescents in a Southern Nigerian State.

**Methods:**

a cross-sectional study design was employed to assess the NCDs´ risk-related knowledge and behaviors among a random multistage sample of 607 students age between 10 and 19 years. Data were collected using an interviewer-administered semi-structured questionnaire adapted from the WHO STEPS questionnaire. Descriptive and inferential analyses of data collected were carried out using the IBM SPSS version 22 software.

**Results:**

the mean age of the students was 14.7 (SD=1.52) years, 57.2% (n=347) of which were females, and 42.8% (n=260) were males. The proportion of students with good overall NCDs risk-related knowledge was 22.7% (n=138). Age, place of residence, family's socioeconomic status, and mother's level of education were significant sociodemographic predictors of good overall NCD risk-related knowledge. Among the students, 66.2% (n=402) self-report inadequate physical activity, 65.7% (n=399) self-report consumption of unhealthy diets, 29.2% (n=177) self-report current alcohol use, and 3.3% (n=20) self-report they were current cigarette smokers.

**Conclusion:**

a significant proportion of the surveyed students had poor overall NCDs risk-related knowledge and engaged in NCDs risk behaviors. The relevant stakeholders concern with prevention of NCDs in government and non-governmental organizations should target adolescents in NCD control strategies in the study setting.

## Introduction

Non-communicable diseases (NCDs) are a group of heterogeneous non-infectious, and not transmissible chronic conditions [1]. They are costly and highly prevalent worldwide, making their prevention a public health priority [2,3]. The global burden of NCDs is substantial as it significantly contributes to poor health and inequalities in health [4]. The costs of NCDs in human, social and economic terms´ are largely preventable and avoidable [3,4]. Unfortunately, about seventy percent of global deaths annually are NCDs-related, with a large proportion occurring prematurely [1,5]. The enormous morbidity and mortality accrued to NCDs occur disproportionately in low- and middle-income countries (LMICs) [1,3,5]. Over sixty percent of NCD-related deaths occur in LMICs [1]. The reasons for the glaring inequity in NCDs-related burden distribution are multiple. They include poverty, weak health systems, rapidly growing populations, ignorance of NCDs-related risk factors and increasing adoption of unhealthy lifestyle choices [6-8]. Non-communicable diseases are sometimes described as lifestyle diseases because many risk factors are behavioral and modifiable. Behaviors like physical inactivity, tobacco smoking, harmful use of alcohol, and consumption of an unhealthy diet rich in trans-fat and dietary salt but low in fresh fruits and vegetables are important modifiable ones [7,9]. These behavioural risk factors are particularly patterned during adolescence, which is a significant phase in the life cycle of NCDs [10]. Being a chronic disease, the latency period of NCDs is long, and the effects of engaging in NCD risk-related behaviors may not be immediately apparent. Thus, the adoption of healthy behavior during the adolescence period is a critical NCDs-preventive strategy [10]. Public health interventions that reduce risky behaviors such as tobacco smoking, harmful use of alcohol, consumption of unhealthy food, and physical inactivity among adolescents are beneficial across the life course and will consequently reduce the burden of NCDs when they eventually become adults [10]. In Nigeria, over one-fifth of the population are adolescents (persons aged between 10 and 19 years) [11]. This large population of adolescents in Nigeria calls for action by the relevant stakeholders in the health and education sectors to implement school-based NCDs-preventive interventions. This study was conducted to assess the level of NCDs´ risk-related knowledge, the prevalence of NCDs´ risk behaviors, and the sociodemographic predictors of NCDs´ risk-related knowledge and behaviors among in-school adolescents in Delta State, Nigeria.

## Methods

**Study setting, design, and participants:** the study setting was at nine secondary schools randomly selected across the three senatorial districts of Delta State in the oil-rich Niger Delta region of Nigeria. Delta State is a culturally diverse State with a total of 758 secondary schools with a total population of 262,242 students distributed across the three senatorial districts [12]. The study design was a school-based cross-sectional survey carried out between December 2021 and May 2022 among a random multistage sample of apparently healthy students aged between 10 and 19 years enrolled in secondary schools in Delta State, Nigeria. The Health Research Ethics Committee (HREC), Delta State University Teaching Hospital provided ethical clearance for the study (HREC/PAN/2021/016/0326). The study conducted followed relevant guidelines and regulations. The authorities of the selected schools provide access permission before the commencement of the study. Participation in the survey was without coercion or inducement. The right to withdraw participation in the survey without untoward consequences was made known to the study participants and their parents/guardians. The study participants and their parents/guardians provided assent and written informed consent, respectively.

**Sample size determination and sampling procedure:** fisher's formula was employed to determine the minimum sample size. Because it was impossible to precise the extent of the various NCD-related risk behaviors in secondary school students in Nigeria, the prevalence was set at 50%. Based on a prevalence of 50%, an error margin of 5% and a standard normal variate at a 95% confidence level, the minimum sample size was estimated at 384 students. However, 607 students participated in the study. Sample selection was in two stages. In the first stage, a table of random numbers was used to select nine secondary schools (three per senatorial district) from a sampling frame of 758 secondary schools in Delta State [12]. In the second stage, apparently healthy students between 10 and 19 years enrolled in the nine selected secondary schools, who gave assent and whose parents/guardians gave informed consent to participate in the study were proportionately allocated into sex strata by their class levels to allow for adequate representation. A table of random numbers was then used to randomly select students from the register in each class level by sex strata. Students who were not within the age bracket of 10 and 19 years were excluded from the sampling process.

**Study instrument and data collection:** data were collected using an interviewer-administered semi-structured questionnaire adapted from the WHO STEPS questionnaire [13]. The questionnaire comprised three sections which assessed the sociodemographic characteristics, NCDs risk-related knowledge and behaviors of the students.

**Outcome and independent variables:** the outcome variables were NCDs risk-related knowledge and behaviors among the students. Knowledge of NCDs risk factors (behavioral and biomedical) was assessed with 11 questions on a 3-Likert scale (agree, disagree, not sure). Each correct response scored one, and every wrong response was zero. There was a maximum of 11 points on the NCDs' risk-related knowledge. Scores of 6 to 11 points were categorized as good overall knowledge, while a score of 0 to 5 points was categorized as poor overall knowledge. NCDs risk-related behaviors were assessed by questions on cigarette smoking, alcohol consumption, diet/nutritional habits, and physical activity habits. Students who self-report that they currently smoke cigarettes were considered as smokers, while those who previously smoked or never smoked cigarettes were considered as non-smokers. Students who self-report that they currently consume alcohol were considered as consumers of alcohol. In contrast, those who previously consumed alcohol or who have never consumed alcohol were considered as non-consumers of alcohol. Students who self-report a daily intake of fewer than five servings of fruits/vegetables (raw or cooked) or regular high intake of saturated fat or sugary meals were categorized as consumers of unhealthy diets. Students who self-report less than 150 minutes/week of moderate to vigorous-intensive physical activity (walking, recreational exercise, cycling) were considered to have inadequate physical activity [14]. The independent variables include age, sex, place of residence, family socioeconomic status and mothers´ educational attainment. Family socioeconomic status was determined using the scoring scheme for classifying socioeconomic status in Nigeria. The scoring scheme takes into cognisance both parents' educational attainment and occupation [15].

**Statistical analyses:** data generated was analyzed using the IBM SPSS version 22 software. Descriptive and inferential analysis of data collected was carried out. Categorical variables were summarized as frequencies and percentages (summarized data were presented in tables and figures). Bivariate and multivariate analysis using Pearson´s chi-square and binary logistic regression respectively was carried out. The statistical significance for both bivariate and multivariate analysis was set at p < 0.05. The 95% confidence intervals (CI) and p-values obtained were reported in two tail form and statistical significance determined at p-value less than 0.05. The binary logistic regression was performed to identify predictors of the outcome variables of interest (NCDs risk-related knowledge and behaviors). All variables significant during bivariate analysis using Pearson´s chi-square at a p-value < 0.2 were entered stepwise into the binary regression model to obtain the adjusted odds' ratio (AOR) of each factor on the outcome variable at 95% confidence interval. The model fitness was measured by the Hosmer-Lemeshow test. The statistical significance of the models (p-value ≥0.776) revealed that the binary logistic regression models (with independent variables included) were good fit to the data.

## Results

The total number of students included in this study was 607, all of whom were included in the analyses.

**Sociodemographic characteristics:** the mean age of the students was 14.7 (SD = 1.52) years, 63.3% (n = 384) of which were aged 14-16 years, 24.2% (n=147) were aged 11-13 years, and 12.5% (n=76) were aged 17-19 years. Their sex distribution revealed that 57.2% (n=347) were female students, while 42.8% (n=260) were male students. The highest proportion of the students were urban residents (56.2%: n=341), compared to those who were rural residents (43.8%: n=266). The highest proportion of the students' families belonged to the middle socioeconomic stratum (68.7%: n=417), compared to those whose families belonged to the lower (18.6%: n=113) and upper (12.7%: n=77) socioeconomic stratum (SES) respectively. The highest proportion of the students' mothers had secondary education (55.8%: n = 339) compared to those whose mothers had primary education (24.1%: n=146) and (20.1%: n=122), respectively (Table 1).

**Table 1:** sociodemographic characteristics of the surveyed adolescents

Variables	Categories	N= 607 (%)
**Age (years)**		
	10 - 13	147 (24.2)
	14 - 16	384 (63.3)
	17 - 19	76 (12.5)
**Mean age ± SD (years)**	14.7±1.52 years	
Sex		
	Male	260 (42.8)
	Female	347 (57.2)
**Place of residence**		
	Urban	341 (56.2)
	Rural	266 (43.8)
**Family SES**		
	Upper	77 (12.7)
	Middle	417 (68.7)
	Lower	113 (18.6)
**Mothers’ education**		
	Primary	146 (24.1)
	Secondary	339 (55.8)
	Tertiary	122 (20.1)

* SES: socioeconomic stratum

**Figure 1:**
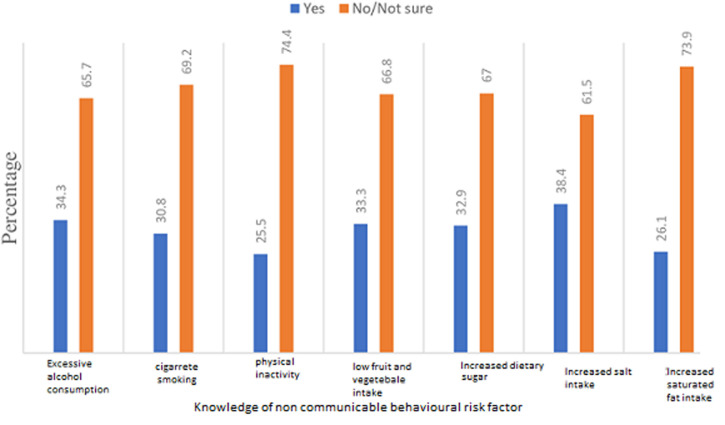
knowledge of non-communicable diseases behavioural risk factors among the surveyed adolescents

**Figure 2:**
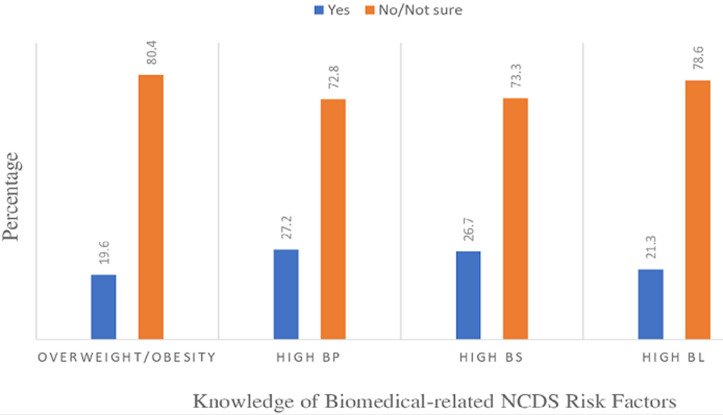
knowledge of biomedical-related non-communicable diseases risk factors among the surveyed adolescents

**Knowledge of NCDs behavioural and biomedical risk factors:** the proportion of students who had good overall NCDs risk-related knowledge was 22.7% (n=138), compared to those who had poor overall NCDs risk-related knowledge (77.3%: n=469) (Table 2). The knowledge of the behavioural and biomedical risk factors among the students was well below average across all the domains assessed (Figure 1, Figure 2). There was a statistically significant age difference (χ^2^= 7.79; df = 2; P = 0.02), urban-rural difference (χ^2^= 5.93; df = 1; P = 0.02), family SES difference (χ^2^= 21.69; df = 2; P < 0.001), and mothers' educational level difference (χ^2^= 13.98; df = 2; P = 0.001) in the level of NCDs risk-related knowledge among the students (Table 2). The students aged 17-19 years (AOR=2.06; 95% CI: 1.53 - 6.09) had two-fold increased odds of having good overall NCDs risk-related knowledge compared to those aged 10-13 years. Students who were rural residents (AOR=0.53; 95% CI: 0.34- 0.83) had 47% decreased odds of having good overall NCDs risk-related knowledge compared to urban residents. Students whose family belonged to the upper SES (AOR=2.32; 95% CI: 1.30-5.28) had two-fold increased odds of having good overall NCDs risk-related knowledge compared to those whose family belonged to the lower SES. Students whose mothers had tertiary education (AOR=2.44; 95% CI: 1.22-5.93) had two-fold increased odds of having good overall NCDs risk-related knowledge compared to those whose mothers had primary education.

**Table 2:** predictors of good overall non-communicable diseases risk-related knowledge among the surveyed adolescents

Variables	Level of knowledge		Bivariate analysis (Chi-square)	Regression analysis adjusted odds' ratio (95% CI)
	Good n=138 (22.7%)	Poor n=469 (77.3%)		
**Age (years)**				
10 - 13	23 (15.6)	124 (84.4)	χ2 = 7.79; df = 2; P=0.02	1
14 - 16	91 (23.7)	293 (76.3)		1.69 (0.96 – 2.97)
17 - 19	24 (31.6)	52 (68.4)		2.06 (1.53-6.09)
**Sex**				
Male	55 (21.2)	205 (78.8)		-
Female	83 (23.9)	264 (76.1)		
**Place of residence**			χ2 = 5.93; df = 1; P = 0.02	
Urban	90 (26.4)	251 (73.6)		1
Rural	48 (18.0)	218 (82.0)		0.53 (0.34 – 0.83)
**Family SES**				
Upper	33 (42.9)	44 (57.1)	χ2 = 21.69; df = 2; P < 0.001	2.32 (1.30--5.28)
Middle	78 (18.7)	339 (81.3)		0.99 (0.41-2.41)
Lower	27 (23.9)	122 (76.1)		1
**Mothers’ education**				
Primary	26 (17.8)	120 (82.2)	χ2 = 13.98; df = 2; P = 0.001	1
Secondary	69 (20.4)	270 (79.6)		1.64 (0.97- 2.75)
Tertiary	43 (35.2)	79 (64.8)		2.44 (1.22 - 5.93)

*SES: socioeconomic status

**Figure 3:**
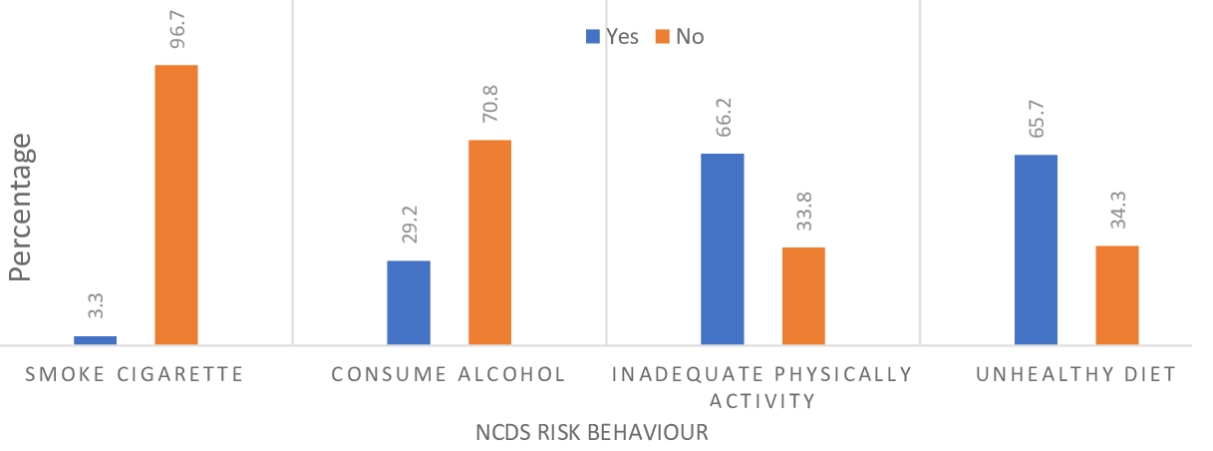
self-reported non-communicable diseases risk behaviours among the surveyed adolescents

**NCDs risk-related behaviours:** the proportion of students who self-report consumption of unhealthy diets was 65.7% (n=399) compared to those who did not self-report consumption of unhealthy diets (34.3%: n=208) (Figure 3). There was a statistically significant family SES difference (χ^2^= 9.32; df = 2; P = 0.009), and mothers' educational level difference (χ^2^= 10.29; df = 2; P = 0.006) in the proportion of students who self-report consumption of unhealthy diet (Table 3). Students whose families belonged to the lower SES (AOR=2.36; 95% CI: 1.07- 5.24) had a two-fold increased odds of self-reporting consumption of unhealthy diet compared to those whose families belonged to the upper SES (Table 3). The proportion of students who self-report inadequate physical activity was 66.2% (n=402), compared to those who self-report adequate physical activity (33.8%: n=205) (Figure 3). There was a statistically significant age difference (χ^2^= 8.01; df = 2; P = 0.02), urban-rural difference (χ^2^= 17.47; df = 1; P < 0.001), family SES difference (χ^2^= 10.71; df = 2; P = 0.01), and mothers' educational level difference (χ^2^= 6.49; df = 2; P = 0.04) respectively in the proportion of students who self-report inadequate physical activity (Table 4). Students who were rural residents (AOR=1.75; 95% CI: 1.20- 2.54) had a two-fold increased odds of self-reporting adequate physical activity compared to urban residents (Table 4).The proportion of students who self-report current alcohol use was 29.2% (n = 177), compared to those who self-report they were not current alcohol users (70.8%: n=430) (Figure 3). There was a statistically significant age difference (χ^2^ = 15.59; df = 2; P < 0.001), and sex difference (χ^2^= 17.51; df = 1; P < 0.001) in the proportion of students who self-report current alcohol use (Table 5). Students aged 14-16 years (AOR=1.76; 95% CI: 1.06 -3.64), and 17-19 years (AOR=1.52; 95% CI: 1.15-3.10), had two-fold increased odds respectively of self-reporting current alcohol use compared to those aged 10-13 years. Female students (AOR=0.45; 95% CI: 0.31-0.64) had 55% decreased odds of self-reporting that they were current alcohol users compared to their male counterparts (Table 5). The proportion of students who self-report they were current cigarette smokers was 3.3% (n = 20), compared to the proportion who self-report they were not current cigarette smokers (96.7%: n=587) (Figure 3). There was a statistically significant sex difference (χ^2^= 4.15; df = 1; P= 0.04) in the proportion of students who self-report that they were current cigarette smokers (Table 6). Female students (AOR=0.39; 95% C.I: 0.15-0.95) had 61% decreased odds of self-reporting that they were current cigarette smokers compared to their male counterparts (Table 6).

**Table 3:** predictors of consumption of healthy diet among the surveyed adolescents

Variables	Consume healthy diet		Bivariate analysis (Chi-square)	Regression analysis adjusted odds' ratio (95% CI)
	Yes n=208 (34.3%)	No n=399 (65.7%)		
**Age (years)**				
10 - 13	48 (32.7)	99 (67.3)	χ2 = 1.68; df = 2; P = 0.43	
14 - 16	129 (33.6)	255 (66.4)		
17 - 19	31 (40.8)	45 (59.2)		
			χ2 = 1.04; df = 1; P = 0.34	
**Sex**				
Male	95 (36.5)	165 (63.5)		
Female	113 (32.6)	234 (67.4)		
				
**Place of residence**			χ2 = 0.44; df = 1; P = 0.55	
Urban	113 (33.1)	228 (66.9)		-
Rural	95 (35.7)	171 (64.3)		
				
**Family SES**				
Upper	39 (50.6)	38 (49.4)	χ2 = 41.80; df = 2; P < 0.001	1
Middle	158 (37.9)	259 (62.1)		1.33 (0.78 - 2.26)
Lower	11 (9.7)	102 (90.3)		2.36 (1.07 - 5.24)
				
**Mothers’ education**				
Primary	65 (44.5)	81 (55.5)	χ2 = 10.29; df = 2; P = 0.006	1
Secondary	100 (29.5)	239 (70.5)		1.01 (0.54 - 1.89)
Tertiary	43 (35.2)	79 (64.8)		1.59 (0.98 - 2.56)

SES: socioeconomic status

**Table 4:** predictors of physical activity among the surveyed adolescents

Variables	Physical activity		Bivariate analysis (Chi-square)	Regression analysis adjusted odds' ratio (95% CI)
	Adequate n=205 (33.8%)	Inadequate n=402 (66.2%)		
**Age (years)**				
10 - 13	37 (25.2)	110 (74.8)	χ2 = 8.01; df = 2; P = 0.02	1
14 - 16	145 (37.8)	239 (62.2)		1.10 (0.59 - 2.08)
17 - 19	23 (30.2)	53 (69.8)		0.67 (0.39 - 1.16)
**Sex**				
Male	84 (32.3)	176 (67.7)		-
Female	121 (34.9)	226 (65.1)		
**Place of residence**			χ2 = 17.47; df = 1; P < 0.001	
Urban	91 (26.7)	250 (73.3)		1
Rural	114 (42.9)	152 (57.1)		1.75 (1.20 - 2.54)
**Family SES**				
Upper	24 (42.9)	53 (57.1)	χ2 = 10.71; df = 2; P = 0.01	1.86 (0.89 - 2.83)
Middle	128 (18.7)	289 (81.3)		1.59 (0.78 - 2.41)
Lower	53 (23.9)	60 (76.1)		1
**Mothers’ education**				
Primary	59 (40.4)	87 (59.6)	χ2 = 6.49; df = 2; P = 0.04	1
Secondary	100 (29.5)	239 (70.5)		1.46 (0.76-2.82)
Tertiary	46 (37.7)	76 (62.3)		1.56 (0.96 - 2.50)

SES: socioeconomic status

**Table 5:** predictors of alcohol consumption among the surveyed adolescents

Variables	Alcohol consumption		Bivariate analysis (Chi-square)	Regression analysis adjusted odds' ratio (95% CI)
	Yes n=177 (29.2%)	No n=430 (70.8%)		
**Age (years)**				
10 - 13	24 (16.3)	123 (83.7)	χ2 = 15.59; df = 2; P < 0.001	1
14 - 16	129 (33.6)	255 (66.4)		1.76 (1.06 - 3.64)
17 - 19	24 (31.6)	52 (68.4)		1.52 (1.15 - 3.10)
			χ2 = 17.51; df = 1; P < 0.001	
**Sex**				
Male	99 (38.1)	161 (61.9)		1
Female	78 (22.5)	269 (77.5)		0.45 (0.31 - 0.64)
				
**Place of residence**			χ2 = 2.31; df = 1; P = 0.13	
Urban	91 (26.7)	250 (73.3)		-
Rural	86 (32.3)	180 (67.7)		
				
**Family SES**				
Upper	19 (24.7)	58 (75.3)	χ2 = 1.25; df = 2; P = 0.54	
Middle	127 (30.5)	290 (69.5)		-
Lower	31 (27.4)	82 (72.6)		
				
**Mothers’ education**				
Primary	37 (25.3)	109 (74.7)	χ2 = 4.04; df = 2; P = 0.13	
Secondary	110 (32.4)	229 (67.6)		-
Tertiary	30 (24.6)	92 (75.4)		

SES: socioeconomic status

**Table 6:** predictors of cigarette smoking among the surveyed adolescents

Variables	Smoke cigarette		Bivariate analysis (Chi-square)	Regression analysis (95% CI)
	Yes n=20 (3.3%)	No n=587 (96.7%)		
**Age (years)**				
10 - 13	3 (2.0)	144 (98.0)	*χ2 = 1.66; df = 2; P = 0.44	
14 - 16	13 (3.4)	371 (96.6)		-
17 - 19	4 (5.3)	72 (94.7)		
			χ2 = 4.15; df = 1; P = 0.04	
**Sex**				
Male	13 (5.0)	247 (95.0)		1
Female	7 (2.0)	340 (98.0)		0.39 (0.15 - 0.95)
				
**Place of residence**			χ2 = 0.12; df = 1; P = 0.73	
Urban	12 (3.5)	329 (96.5)		-
Rural	8 (3.0)	258 (97.0)		
				
**Family socioeconomic status**				
Upper	4 (5.2)	73 (94.8)	*χ2 = 1.12; df = 2; P = 0.57	
Middle	12 (2.9)	405 (97.1)		-
Lower	4 (3.5)	109 (96.5)		
				
**Mothers’ education**				
Primary	6 (4.1)	140 (95.9)	*χ2 = 0.57; df = 2; P = 0.75	
Secondary	11 (3.2)	328 (96.8)		-
Tertiary	3 (2.5)	119 (97.5)		

## Discussion

The overall NCDs´ risk-related knowledge among a significant proportion of the students was generally poor. An assessment of all the NCDs´ risk-related knowledge domains tested among them were below average. This is a worrisome development which calls for urgent intervention of all stakeholders in the education and health sectors involved in adolescent health in Nigeria. This is important because risk behaviors that promote NCDs thrive in the background of poor risk-related knowledge [16]. The adolescence period is a critical transitory stage in life between childhood and adulthood. It is a period of life characterized by the development of mental capacity, and significant physical, emotional, and behavioral changes. Potentially, the patterns of NCDs risk behaviors are shaped during this period and tend to persist in adulthood [10]. Therefore, the need to equip adolescents with the knowledge and skills to adopt healthy behaviors and choices before they become adults cannot be overemphasized. Overall, only one-fifth of the students had good overall NCDs´ risk-related knowledge. This observation corroborates with findings from previous studies conducted in Nigeria [17,18], and elsewhere in India [19], Sri Lanka [20], and Thailand [21] which have revealed similar poor levels of NCDs risk-related knowledge among in-school adolescents. Knowledge impacts health, and it is in turn determined by several factors that are sometimes complex and interrelated. In this study, sociodemographic factors such as age, place of residence, family's socioeconomic status and mothers' education were the predictors of good overall NCDs risk-related knowledge among the students. Indeed, age, work and living environment, family income, and mother's educational attainment are well-described social determinants of health [22]. The likelihood of having good overall NCDs risk-related knowledge was two-fold higher respectively among students who were older (late adolescents; 17-19 years), whose family belonged to the upper socioeconomic class, and whose mothers had tertiary education. Furthermore, students who were rural dwellers had forty-seven percent decreased likelihood of having good overall NCDs risk-related knowledge compared to their urban counterpart. It is not unlikely that the older students surveyed had amassed more knowledge over time as most of them were in higher school grades. It is also not unlikely that the students living in urban areas, whose family belonged to the upper socioeconomic stratum and whose mothers had tertiary education (more than 12 years of formal education) may have access to diverse sources of information aside from school-based health education, including the internet and other forms of electronic media, and thus acquire more knowledge. However, this study did not assess the sources of NCDs related information among the students.

In addition to the poor overall NCDs´ risk-related knowledge, a high rate of risk behaviors that favor the development of NCDs was observed among the students. About two-thirds of the students consumed unhealthy diets (majorly low in fruits and vegetables). This observation corroborates with findings from a previous study in Ghana conducted among in-school adolescents [23]. Correspondingly, a meta-analysis of seventy-two countries drawn from all the World Health Organization (WHO) regions, and a United Nations Children's Fund (UNICEF)report respectively revealed high consumption of unhealthy diets (low in fruits and vegetables, and high in sugar and saturated fat) [24,25]. Consumption of unhealthy diets among the students differed by family socioeconomic status. The likelihood of consuming unhealthy diets was two-fold higher among students whose family belonged to the lower socioeconomic stratum. Evidence have shown that individual choices of diet are constrained by socioeconomic factors including environmental, political, and cultural factors [4]. Therefore, affordability of healthy diet is a critical factor for low incomes families in all countries. Consequently, low incomes families consume lower quantities of fruit and vegetables than high income families [4]. Similarly, about two-thirds of the students were physically inactive. This was not surprising as over the past decades, sub-Saharan African adolescents have transitioned mainly from being quite physically active to being sedentary [26]. Walking as a mode of transportation is often replaced with riding in motorized vehicles, and video/computer games have largely replaced engagement in outdoor sporting activities. Also, increased screen time (spent on computers, television and mobile phones) has adversely affected physical activity among adolescents. Other studies among adolescents outside the African continent have reported a preponderance of physical inactivity [27,28]. Indeed, according to the World Health Organization (WHO), more than 80% of global adolescents lead sedentary lifestyles [29]. In this study, students who were rural dwellers were more physically active compared to their urban counterpart. The likelihood of being physically active was two-fold higher among students who were rural dwellers.

In this study, about a third of the students admit to current alcohol use. The prevalence of current alcohol use observed among the students falls within the range previously reported among adolescents in Nigeria [30,31]. Alcohol consumption, a self-harming behavior, is rife among adolescents [30-33]. Despite policies against underage drinking, alcohol consumption remains the most common substance of abuse among young people in Nigeria [34,35]. Alcohol consumption was significantly associated with age and sex. Students aged 14-16 years (mid-adolescents) and 17-19 years (late adolescents) had 2-fold higher odds of consuming alcohol compared with early adolescents (10-13 years). Furthermore, a significantly lower proportion of female students admit current alcohol use, and they had fifty-five percent decreased likelihood of alcohol use compared to their male counterpart. Previous studies had similarly reported male preponderance in alcohol consumption among adolescents in Nigeria [32,33]. It is noteworthy that some studies report no sex difference in the prevalence of alcohol consumption among adolescents unlike adult drinkers with consistent male preponderance [31,36]. Tobacco smoking, another harmful social habit, was reported by 3.3% of the students. The significant sociodemographic predictor for tobacco smoking in this study was sex, with female students having sixty-one percent decreased likelihood of smoking cigarettes compared to their male counterpart. The preponderance of male smokers in this study corroborates with the findings of a systematic review which revealed a higher prevalence of male adolescent smokers in Nigeria [37]. The findings of our study should be interpreted considering the following strengths and limitations. The main strength of our study is that it targeted a relatively larger population of in-school adolescents. However, our study has some limitations: firstly, the self-report nature of NCDs risk behaviors by the students leaves room for reporter bias. Secondly, this study employed a cross-sectional descriptive design; thus, it is not possible to determine either causality or directionality of the variables analyzed.

## Conclusion

A significant proportion of the students in this study had poor overall NCDs risk-related knowledge. Age, place of residence, family's socioeconomic status, and mother's level of education were significant sociodemographic predictors of good overall NCDs risk-related knowledge. In addition, a high rate of risk behaviors that favor the development of NCDs was observed among the students. There is need for concerted efforts by the relevant stakeholders that are concern with prevention of NCDs in government and non-governmental organizations to prioritize in-school adolescents in NCD control strategies in the study location. This calls for urgent implementation of interventions at all ecological levels that will improve adolescents' NCDs risk-related knowledge and equip them with the skills to adopt healthy lifestyles and choices. This consequently will deter their engagement in unhealthy behaviors.

### What is known about this topic

- *Non-communicable diseases are a significant public health problem world-wide;*
- *Adolescence period is a significant phase in the life cycle of non-communicable diseases*.

### What this study adds

- *Poor levels of non-communicable diseases risk-related knowledge among in-school adolescents;*
- *High rates of non-communicable diseases risk-related behaviours among in-school adolescents*.

## References

[1] World Health Organisation (WHO) Noncommunicable diseases.

[2] Murphy A, Palafox B, Walli-Attaei M, Powell-Jackson T, Rangarajan S, Alhabib KF (2020). The household economic burden of non-communicable diseases in 18 countries. BMJ Glob Health.

[3] NCD Countdown 2030 collaborators (2018). NCD Countdown 2030: worldwide trends in non-communicable disease mortality and progress towards Sustainable Development Goal target 4. Lancet.

[4] Marmot M, Bell R (2019). Social determinants and non-communicable diseases: time for integrated action. BMJ.

[5] GBD 2017 Causes of death Collaborators (2018). Global, regional, and national age-sex-specific mortality for 282 causes of death in 195 countries and territories 1980-2017: A systematic analysis for the Global Burden of Disease Study 2017. Lancet.

[6] Yuyun MF, Sliwa K, Kengne AP, Mocumbi AO, Bukhman G (2020). Cardiovascular Diseases in sub-Saharan Africa Compared to High-Income Countries: An Epidemiological Perspective. Glob Heart.

[7] Allen L, Williams J, Townsend N, Mikkelsen B, Roberts N, Foster C (2017). Socioeconomic status and non-communicable disease behavioural risk factors in low-income and lower-middle-income countries: a systematic review. Lancet Glob Health.

[8] Pullar J, Allen L, Townsend N, Williams J, Foster C, Roberts N (2018). The impact of poverty reduction and development interventions on non-communicable diseases and their behavioural risk factors in low and lower-middle-income countries: A systematic review. PLoS One.

[9] GBD 2015 Risk Factors Collaborators (2016). Global, regional, and national comparative risk assessment of 79 behavioural, environmental and occupational, and metabolic risks or clusters of risks 1990-2015: a systematic analysis for the Global Burden of Disease Study 2015. Lancet.

[10] World Health Organization Global accelerated action for the health of adolescents (AA-HA): guidance to support country implementation.

[11] Cortez R, Saadat S, Marinda E, Oluwole O (2015). Adolescent sexual and reproductive health in Nigeri. Health, nutrition and population global practice knowledge brief.

[12] Delta State Government Delta State Government.

[13] World Health Organization WHO STEPS surveillance manual: the WHO STEPwise approach to chronic disease risk factor surveillance/non-communicable diseases and mental health, World Health Organization.

[14] James S National Survey for Wales, 2019-20: adult lifestyle. Policy commons.

[15] Ibadin MO, Akpede GO (2021). A revised scoring scheme for the classification of socio-economic status in Nigeria. Niger J Paediatr.

[16] Tohi M, Bay JL, Tu'akoi S, Vickers MH (2022). The Developmental Origins of Health and Disease: Adolescence as a Critical Lifecourse Period to Break the Transgenerational Cycle of NCDs-A Narrative Review. Int J Environ Res Public Health.

[17] Shabi IN, Oyewusi FO (2018). Health literacy and internet health information use among in-school adolescents in Osun State, South-West, Nigeria. J Consum Health Internet.

[18] Odusoga OB, Sholeye OO (2023). Sedentary Behaviour Among Male Adolescents in Sagamu, Southwest Nigeria. Community Health Equity Res Policy.

[19] Bassi S, Bahl D, Harrell MB, Jain N, Kandasamy A, Salunke SR (2021). Knowledge, attitude, and behaviours on diet, physical activity, and tobacco use among school students: A cross-sectional study in two Indian states. F1000Res.

[20] Gamage AU, Jayawardana PL (2017). Knowledge of non-communicable diseases and practices related to healthy lifestyles among adolescents, in state schools of a selected educational division in Sri Lanka. BMC Public Health.

[21] Lorga T, Aung MN, Naunboonruang P, Junlapeeya P, Payaprom A (2013). Knowledge of communicable and noncommunicable diseases among Karen ethnic high school students in rural Thasongyang, the far northwest of Thailand. Int J Gen Med.

[22] Braveman P, Gottlieb L (2014). The social determinants of health: it´s time to consider the causes of the causes. Public Health Rep.

[23] Hormenu T (2022). Dietary intake and its associated factors among in-school adolescents in Ghana. PLoS One.

[24] Beal T, Morris SS, Tumilowicz A (2019). Global pattern of adolescent fruit, vegetable, carbonated soft drink, and fast-food consumption: a meta-analysis of global school-based student health surveys. Food Nutr Bull.

[25] United Nations Children´s Fund The State of the World´s Children 2019, Children, Food and Nutrition: Growing well in a changing world.

[26] Wachira L Lifestyle Transition towards Sedentary Behaviour among Children and Youth in Sub-Saharan Africa: A Narrative Review.

[27] Dias PJ, Domingos IP, Ferreira MG, Muraro AP, Sichieri R, Gonçalves-Silva RM (2014). Prevalence and factors associated with sedentary behavior in adolescents. Rev Saude Publica.

[28] Nascente FMN, Jardim TV, Peixoto MdRG, Carneiro CdS, Mendonca KL, Povoa TIR (2016). Sedentary lifestyle and its associated factors among adolescents from public and private schools of a Brazilian State capital. BMC Public Health.

[29] Guthold R, Stevens GA, Riley LM, Bull FC (2020). Global trends in insufficient physical activity among adolescents: a pooled analysis of 298 population-based surveys with 1•6 million participants. Lancet Child Adolesc Health.

[30] Alex-Hart BA, Opara PI, Okagua J (2015). Prevalence of alcohol consumption among secondary school students in Port Harcourt, Southern Nigeria. Niger J Paed.

[31] Mbadiwe N, Adikaibe BE, Achor J, Okoye I, Onodugo O, Anyim O (2021). Alcohol Use in selected low-income neighbourhoods in Enugu, Southeast Nigeria. Ann Med Health Sci Res.

[32] Granville-Garcia AF, Clementino MA, Gomes MNC, Firmino RT, Ribeiro GLA, Siqueira MBLD (2014). Alcohol consumption among adolescents. Ciênc Saúde Coletiva.

[33] Hormenu T, Hagan Jnr JE, Schack T (2018). Predictors of alcohol consumption among in-school adolescents in the Central Region of Ghana: a baseline information for developing cognitive-behavioural interventions. PLoS One.

[34] Dumbili EW (2014). The politics of alcohol policy in Nigeria: a critical analysis of how and why brewers use strategic ambiguity to supplant policy initiatives. J Asian Afr Stud.

[35] Gureje O, Degenhardt L, Olley B, Uwakwe R, Udofia O, Wakil A (2007). A descriptive epidemiology of substance use and substance use disorders in Nigeria during the early 21^st^ century. Drug Alcohol Depend.

[36] Kuntsche E, Wicki M, Windlin B, Roberts C, Gabhainn SN, van der Sluijs W (2015). Drinking motives mediate cultural differences but not gender differences in adolescent alcohol use. J Adolesc Health.

[37] Oyewole BK, Animasahun VJ, Chapman HJ (2018). Tobacco use in Nigerian youth: A systematic review. PLoS One.

